# The Incidence and Types of Physical Contact Associated with Body Checking Regulation Experience in 13–14 Year Old Ice Hockey Players

**DOI:** 10.3390/ijerph13070668

**Published:** 2016-07-07

**Authors:** Claude Goulet, Thierry-Olivier Roy, Luc Nadeau, Denis Hamel, Kristine Fortier, Carolyn A. Emery

**Affiliations:** 1 Department of Physical Education, Faculty of Education, Laval University, Québec, QC G1V 0A6, Canada; luc.nadeau@fse.ulaval.ca (L.N.); kristine.fortier@fse.ulaval.ca (K.F.); 2 Department of Kinesiology, Faculty of Medicine, Laval University, Québec, QC G1V 0A6, Canada; to.roy@icloud.com; 3 Québec Public Health Institute, Québec, QC G1V 5B3, Canada; denis.hamel@inspq.qc.ca; 4 Sport Injury Prevention Research Centre, Faculty of Kinesiology, University of Calgary, Calgary, AB T2N 1N4, Canada; caemery@ucalgary.ca; 5 Alberta Children’s Hospital Research Institute, University of Calgary, Calgary, AB T2N 4N1, Canada; 6 Department of Pediatrics and Community Health Sciences, Cumming School of Medicine, Faculty of Medicine, University of Calgary, Calgary, AB T2N 1N4, Canada

**Keywords:** ice hockey, behaviours, physical contact, body checking, prevention, athletic injuries

## Abstract

Background: Ice hockey has one of the highest sport participation and injury rates in youth in Canada. Body checking (BC) is the predominant mechanism of injury in leagues in which it is permitted. The objectives of this study were to determine whether the incidence and types of physical contact differ for Bantam players (aged 13–14 years) who were exposed to BC at Pee Wee level (aged 11–12 years) in Calgary, Alberta versus Bantam players who were not exposed to BC at Pee Wee level in Québec City, Québec. All teams were exposed to BC at bantam level; Methods: A cohort study was conducted in Québec City and Calgary. Sixteen games for Calgary and 15 for Québec City were randomly selected and analysed with a validated observation system to quantify five intensities of physical contact and to observe different types of physical contact such as slashing and holding; Results: A total of 5610 incidences of physical contact with the trunk and 3429 other types of physical contact were observed. Very light intensity trunk contact was more frequent in Calgary (adjusted incidence RR (ARR): 1.71; 95% CI: 1.28–2.29). Holding (ARR: 1.04; 95% CI: 1.02–1.07) and slashing (ARR: 1.38; 95% CI: 1.07–1.77) were more frequent in Calgary; Conclusion: Results suggest that players’ physical contacts differ between Bantam leagues in which BC was permitted at Pee Wee level and leagues in which it was not permitted until Bantam level.

## 1. Introduction

Ice hockey is a long-standing and beloved tradition in Canada. In 2015, Hockey Canada encompassed 3500 associations, for more than 639,000 players [1]. In the province of Québec, more than 100,500 individuals were registered in Hockey Québec. At the other end of the country, Hockey Alberta counted around 73,500 registered players in the last season [1]. Although ice hockey is one of Canada’s most popular sports, there is an increasing concern about the frequency of ice hockey injuries in youth. Canadian data suggest that ice hockey injuries account for 10% of all youth sport injuries [2,3,4]. Body checking has been associated with 45% to 86% of injuries among youth ice hockey players [2,5,6,7]. Hockey Canada distinguishes body contact and body checking. Body contact is defined as “an individual defensive tactic designed to legally block or impede the progress of an offensive puck carrier. This tactic is the result of a defensive player applying physical extension of the body toward the puck carrier moving in an opposite or parallel direction” [8]. Usually, when such contact occurs, the puck carrier is not significantly affected: the skating momentum may be halted or the direction changed, but the player should be able to continue playing. However, when body checking occurs, in most cases the skating direction is changed and the puck carrier is significantly affected. In this paper, physical contact includes body contact and body checking.

Several studies have demonstrated that body checking is the primary mechanism of ice hockey injury, including concussion [6,9,10,11,12]. It has been observed that when body checking is permitted at Pee Wee level, the injury risk rises considerably. In fact, Emery et al. demonstrated that there were three times more injuries and four times as many concussions when body checking was allowed [13]. Furthermore, a 2012 study in Pee Wee players showed that more lower-intensity physical contacts, hooking and slashing occurred in Québec City, versus more high-intensity physical contacts and pushing in Calgary [14]. It is noteworthy that, in 2013, Québec was the only Canadian province in which body checking was not permitted before Competitive Bantam level.

Body checking is not only the primary mechanism of injury, it also appears to be a vector of violence and intimidation [15,16]. Players often try to instill fear in the most talented opponents so as to inhibit them and increase their own chances of winning the match [15,16]. A collision between two young players skating in opposite directions can be highly detrimental to both their physical and mental health [12,16,17].

The issue of introducing body checking at the Elite Bantam level (13–14 years old) has fuelled significant controversy in Canadian youth ice hockey [18]. In Québec, the decision to start body checking at Bantam level was based largely on a study conducted by the Régnier team, who showed that introduction to body checking at younger ages resulted in more penalties, more injuries, and more aggressiveness in general [17]. Early initiation to body checking can directly impact the game of hockey as well as the type of players who develop into young athletes [16,17]. To these we may add the Policy Statement by the American Academy of Pediatrics, in which it is recommend that body checking be prohibited before the age of 16 years [19]. The Canadian Pediatric Society and some studies confirm that statement and suggest delaying the introduction of body checking in elite male competitive league until players are in Bantam level [10,20].

There are two divergent schools of thought on this issue. The first includes those who believe in early initiation to body checking in order to provide youth with the knowledge and practice that will enable them to perform body checking more effectively and be more comfortable with it. They suggest that when young ice hockey players get used to giving and receiving body checking, there is a protective effect when they get to higher level like Midget (15–16 years) [18]. However, prospective evidence contradicts this opinion and demonstrates that body checking experience does not reduce the risk of overall injury or concussion [21].

The second school of thought suggests that later introduction of body checking also allows young players to concentrate on developing more technical and tactical skills such as shooting, passing, skating, defensive strategies, or power plays [15]. At the Pee Wee level (11–12 years), growth and puberty are far from complete, and far from uniform across players. Thus, according to Régnier, young players show differences in size up to 31.5 centimeters (12.4 inches) and almost 37 kilograms (about 82 pounds) in weight [17]. Given these relatively drastic differences, the risk of injury is great.

When this study was conducted (season 2008–2009), Alberta and Québec had different regulations related to body checking. In Québec, élite players were introduced to a regulation allowing body checking at Bantam level (13–14 years). In Alberta, they were introduced to body checking two years earlier, at Pee Wee level (11–12 years). The different regulations between the two provinces provided a unique opportunity to compare disparities in players’ behaviours related to physical contact in Calgary, Alberta, and Québec City, Québec. The aim of this study was therefore to explore whether these differences in regulation of body checking wielded an impact on the type and intensity of physical contacts in youth ice hockey. The main objective was to determine whether the incidence and types of physical contact differed between Bantam players who were exposed to body checking at Pee Wee level (Calgary), and Bantam players who were not yet exposed to body checking at Pee Wee level (Québec City).

This study should provide those in charge of youth ice hockey skill development across Canada and other countries where ice hockey is popular with objective data to assist them in making informed decisions on the appropriateness of exposing young ice hockey players to body checking. Since the past five years, the sensibility to injury prevention, specifically to concussion prevention, have increased in researchers, sport administrators, and community stakeholders [18]. In Canada and in the United States of America, this increased sensibility and research evidence triggered policy change delaying the age of body checking in youth ice hockey. But, recent studies comparing offensive skills [22], and contact mechanisms [23] of Pee Wee players (11–12 years), before (2007–2008) and after body checking policy change (2013–2014), did not reveal significant changes that would suggest that data of this study are not valid. We have no reason to believe that the situation is different for Bantam players (13–14 years).

## 2. Materials and Methods

This cohort study includes games within one regular season: from January to March 2009. The study population included Bantam ice hockey teams in Calgary and Québec City. One group (Calgary) included players who had been exposed to body checking at Pee Wee level, and the other (Québec City) was made up of players who had not. Although the terms for the competitive categories differed between the two provinces, all teams selected for this study were playing in the upper 30% by competitive level of play in the Bantam League.

**Figure 1:**
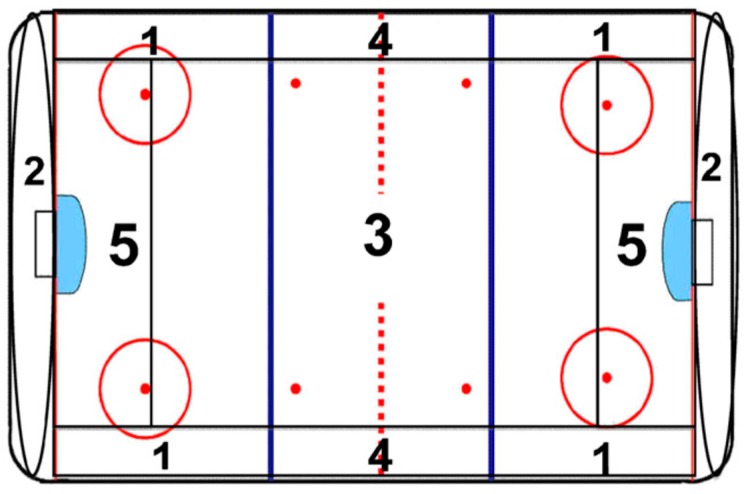
Definition of the zones on the playing surface. Numbers indicate the zones where a physical contact may occur.

Based on the work of Nadeau and his colleagues [24], Malenfant et al. [14] developed and validated an observation system for recording, quantifying, and qualifying incidences of physical contact during an ice hockey game. This system was used in the present study to analyse 31 games randomly selected. Sixteen games were analyzed for the Calgary group and 15 for the Québec City group. All games were recorded on DVD media and analyzed by research assistants, with inter-judge reliability determined at over 90% for each characteristics of physical contact observed. All research assistants were trained by one of the co-authors (LN) with standardized footage of each categories of physical contact until at least 90% of inter-judge reliability was obtained for all types of physical contact. To make sure that inter-judge reliability was maintained at least at 90% for each research assistant, re-test of inter-judge reliability was made at least two times during the period of video analysis. During the video analyses, the research assistants noted specific characteristics of observed physical contacts (Table 1). To account for the different places where the incidences took place, the skating rink was divided into five zones (see Figure 1). Two main types of body contact could occur: executed with the trunk (i.e., shoulders and hips) or executed with a limb or an object (e.g., arm or ice hockey stick). Each types of contact made with the limbs or an object are different and mutually exclusive categories of contact*.* In both cases, other contact data were coded. The researchers also noted whether each physical contact was made by an offensive or defensive player, whether on a puck carrier or not, and whether or not it was deliberate. When the body contact involved a limb or object, the researchers noted the limb or object used. The following other types of physical contact were also recorded: pushing, holding, slashing, and tripping. The consequences of the physical contacts (i.e., penalized or not) were not recorded. In fact, a physical contact could be penalized or not, mainly based on the judgement of the referee on how the impact of the contact interfered with the player sustaining it.

The intensity of physical contacts made with the trunk were rated from 1 to 5. Level 1 refers to very light physical contact between players who are not moving forward. Players routinely battle for the puck along the boards or after a face-off. With this type of contact, it is sometimes difficult to distinguish between the puck carrier and the non-puck carrier, or to determine whether the player is offensive or defensive. Level 2 refers to light physical contact between two players skating in the same direction. The intention is to impede the progress of the opposing player, who is slightly or not at all affected by the contact. Level 3 refers to moderate physical contact between two players skating in the same direction. The intention is to impede the progress of the opposing player, who is moderately or totally affected by the contact. Level 4 physical contact occurs when a player applies a forceful physical extension of the body to an opposing player, who is usually skating in the opposite direction. The opposing player is moderately or totally affected by the contact. It corresponds to a heavy intensity physical contact. Level 5 physical contact occurs when a player deliberately extends toward an opposing player, who is usually skating in the opposite direction, in order to initiate contact and hit with excessive force. The intention is more than simply to impede the progress of the opponent, who is totally affected by the contact. Physical contacts of levels 4 and 5 correspond to body checking as defined by Hockey Canada [8].

SAS version 9.2 (SAS Institute Inc., Cary, NC, USA) was used for the statistical analyses. Incidence rates were based on number of physical contacts per team-game. Crude and adjusted incidence rate ratio (RR) based on multivariate Poisson regression were used to compared games played in Calgary and Québec City. To obtain valid estimates, RRs were adjusted for period of play (first, second, third), zone on the playing surface (Figure 1), and score difference (0–1 goal, 2 or more). The data were analysed using Generalized Estimating Equations (GEE) accounting for potential cluster effect (games). Over dispersion of data were also considered, using a Negative Binomial Distribution. Significance was based on α < 0.05. Approval was granted by the ethical research committees of Laval University (approval number: 2007-134 A-1) and University of Calgary (ETHICS ID 20252).

## 3. Results

A total of 5610 physical contacts using the trunk (rate: 90.9/team-game) and 3429 other types of contact (rate: 55.3/team-game) were observed. As presented in Table 2, the only difference observed in terms of contact intensity was for level 1, which was observed more frequently in Calgary than in Québec City games (ARR: 1.71; 95% CI: 1.28–2.29). More defensive players made physical contacts in Calgary than in Québec City (ARR: 1.28; 95% CI: 1.03–1.58), more puck carriers received body contacts in Calgary than in Québec City (ARR: 1.47; 95% CI: 1.02–2.12), and more non deliberate physical contacts were observed in Calgary than in Québec City (ARR: 1.89; 95% CI: 1.03–3.46).

The overall rates for physical contact with a limb or object did not differ between leagues in the two cities. However, when contact type was analyzed, certain differences appeared. Calgary players slashed (ARR: 1.38; 95% CI: 1.07–1.77) or held (ARR: 1.04; 95% CI: 1.02–1.07) more often than Québec City players.

The rink areas where the most contacts occurred were, in descending order, zone 2 (1518), zone 3 (1366), and zone 1 (1365). Zones 2 and 1, which include the entire board lengths for both the offensive and defensive ends, are areas where effective physical contact is most required. A large difference was observed in contact frequency in front of the goal (zone 5), where Calgary players made contact almost twice as often as Québec City players (616 versus 324).

## 4. Discussion

Based on the findings, we note significant differences between the two cohorts of Bantam players that were exposed or not to a regulation allowing body checking, in terms of physical contacts using the trunk, intensity of physical contacts using the trunk, and number of contacts categorized as slashing or holding. Thus, Calgary players made more level 1 contacts, but no significant inter-city difference was found in terms of higher-intensity contacts (see Table 2). Therefore, given the rates of level 1 physical contacts, which are less intense, it is difficult to conclude that the Calgary teams played a rougher game.

Although the level 3 to 5 contacts did not differ between the two cohorts, two variables were statistically different in the category “other physical contacts.” The frequencies of physical contacts categorized as slashing and holding were significantly higher in Calgary games (Table 2). This suggests that the game involves more physical contacts frequently penalized when players have been exposed to and are used to body checking at Pee Wee level. However, aside from the fact that body checking was permitted, we should remind the reader that holding is generally considered a tactic to impede the opposing player’s progress, like other types of physical contact [8]. It follows that all the types of physical contact (pushing, holding, slashing, and tripping) should be viewed as attempts to learn defensive skills, and should not be considered as meriting penalties at first, unless they cross the line of legality.

According to these results, exposure to body checking regulation at Pee Wee level could result in better assimilation and use of body checking in game situations. In fact, the defensive players on Calgary teams used physical contacts more frequently on the puck carrier (Table 2), which is considered the proper use of body contact and body checking [8]. Thus, for defensive players, the goal is to impede the puck carrier’s progress. This suggests that players in Calgary leagues can execute body checking more effectively. Even though body checking effectiveness was not assessed, the fact that physical contacts were executed more often on a puck carrier, and by a defensive player, supports the idea of better assimilation of body contact and body checking skills.

The limitations of our study should be discussed. First, there is no reason to believe that the sample of analysed games (*n* = 31), nor the total of physical contact observed (*n* = 9039) were not representative of the type of ice hockey played in Calgary and Québec City for teams in the top 30% by level of play. The limited number of games observed on the other hand could reduce the power of the analyses. Nevertheless, interesting differences were found related to the characteristics of the physical contacts made with the trunk and the other types of physical contact. Furthermore, some physical contacts may not have been observed or recorded by the research assistant because of the poor image quality of the video or physical contacts made in a camera’s dead angle. However, there is little reason to believe that a systematic selection bias associated with those missed actions occurred. The teams were randomly selected and the observers were regularly reviewed by one of the co-authors (LN) to assess the level of agreement between observers on the different categories of physical contact. Moreover, we were not able to measure the intention of the players just before the physical contact occurred and thus we cannot know the players’ intent of the physical contact (i.e. to limit the progression of the opponent or to cause harm). This type of information would be needed in an injury prevention perspective. The issue of the game score and the general standing of the teams were unknown. The observation system did not allow for measurement of the impact of these two variables. A better understanding on how the incidence and the types of physical contact evolve during a single game, or during a full season is needed. Finally, the quality of player skill was not assessed despite the fact that a skillful player might play differently than a low skilled player. Therefore, we were not able to report if players in leagues where body checking was permitted at Pee Wee level played differently than those in leagues without body checking at Pee Wee level. Further studies are necessary to compare the skill level of players playing with or without body checking at Pee Wee level. Additionally, since the study was anonymous, it was impossible to link the types of physical contact with injury surveillance data.

## 5. Conclusions

In conclusion, the results suggest that the incidence of low-intensity physical contacts was generally higher at Bantam level in leagues where players were exposed to body checking at Pee Wee level (Calgary). Generally, games in Calgary were also more physically intense, because players slashed and held more often than their counterparts in Québec City. We may conclude that there are some differences between players who have been exposed to body checking at Pee Wee level and players who are not exposed to body checking until Bantam level. The length of exposure to body checking may therefore affect the intensity and types of physical contact during Bantam-level ice hockey games. On the other hand, this did not seem to influence risk of injury [21].

This study provides those in charge of youth ice hockey skill development across Canada and other countries with objective data to assist them in making informed decisions on the appropriateness of exposing young players to body checking. Coaches must know that body checking could lead to higher injury rates due to the higher frequency, intensity and the difference in type of physical contacts and adapt their teaching of body checking accordingly. Trainers should be aware that slashing and holding seems to be more frequent in cities where teams have been exposed to body checking at Pee Wee level.

## Figures and Tables

**Table 1:** Characteristics of the physical contact (PC) observed.

Outcome	Measurements
Type of physical contact	Made with the trunk (Level of intensity 1 to 5)
	Other types of contact made with the limbs or a hockey stick: Slashing, holding, pushing, tripping
Who provide the PC	Defensive player
	Offensive player
	Puck carrier
	Non puck carrier
Intension of the player when doing the PC	Deliberate
	Non deliberate

**Table 2:** Characteristics of the physical contacts (PC) made with the trunk and other PC made by Bantam ice hockey players (13–14 years old) in leagues in Québec City and in leagues in Calgary (2008–2009 season).

Intensity of PC with the Trunk ^1^	Québec City N/Team-game	Calgary N/Team-game	RR (95% CI) ^2^	ARR (95% CI) ^3^	*p*-Value for ARR
All	83.6	100.1	1.19 (1.03–1.39)	1.21 (1.02–1.44)	0.03
1	16.1	28.0	1.74 (1.24–2.43)	1.71 (1.28–2.29)	0.0003
2	24.7	30.1	1.22 (0.93–1.59)	1.24 (1.05–1.62)	0.11
3	19.7	22.4	1.13 (0.88–1.46)	1.13 (0.89–1.43)	0.33
4	18.5	15.2	0.82 (0.61–1.09)	0.82 (0.61–1.11)	0.20
5	4.5	4.5	1.00 (0.63–1.61)	1.00 (0.63–1.61)	0.99
**PC made with the trunk**					
By an offensive player	17.3	16.7	0.97 (0.57–1.66)	0.97 (0.58–1.61)	0.89
By a defensive player	66.3	83.4	1.26 (1.02–1.55)	1.28 (1.03–1.58)	0.02
On a puck carrier	48.0	70.7	1.47 (1.03–2.10)	1.47 (1.02–2.12)	0.03
On a non carrier	35.6	29.5	0.83 (0.47–1.44)	0.73 (0.39–1.31)	0.31
Non-deliberate	1.5	2.84	1.93 (1.06–3.53)	1.89 (1.03–3.46)	0.039
**Other PC**					
All	52.1	60.2	1.16 (0.82–1.63)	1.31 (0.68–2.52)	0.41
Pushing with a limb	36.7	35.0	0.95 (0.60–1.51)	0.94 (0.55–1.63)	0.59
Holding with a hockey stick	1.1	2.4	2.21 (1.45–3.35)	1.04 (1.02–1.07)	0.001
Slashing with a hockey stick	4.7	14.3	3.04 (1.15–8.51)	1.38 (1.07–1.77)	0.012
Tripping	9.6	8.6	0.89 (0.59–1.34)	0.97 (0.86–1.09)	0.59

^1^: Intensity as define in the Materials and Methods Section; ^2^: RR, incidence rate ratio Calgary vs. Québec City; CI, confidence interval; ^3^: ARR, incidence rate ratio Calgary vs. Québec City adjusted for game period, score difference, and zone on the playing surface; CI, confidence interval.
